# The prevalence of self-medication in breastfeeding mothers during the COVID-19 pandemic

**DOI:** 10.34763/jmotherandchild.20222601.d-22-00021

**Published:** 2022-12-21

**Authors:** Samaneh Naseri, Bahareh Bahman Bijari, Fatemeh Dabaghzadeh, Tania Dahesh

**Affiliations:** Herbal and Traditional Medicines Research Center, Kerman University of Medical Sciences, Kerman, Iran; Department of Pediatrics, Afzalipour Medical Center, Kerman, Iran; Department of Clinical Pharmacy, Faculty of Pharmacy, Kerman University of Medical Sciences, Kerman, Iran; Modeling in Health Research Center, Institute for Futures Studies in Health, Kerman University of Medical Sciences, Kerman, Iran

**Keywords:** Breastfeeding, information, mothers, self-medication, side effects

## Abstract

**Background:**

Self-medication is an important health and social issue, especially for women due to critical periods such as pregnancy and lactation. Accordingly, this study aimed to investigate the use of nonprescribed medications by lactating mothers visiting health centers affiliated to Kerman University of Medical Sciences during the COVID-19 pandemic.

**Material and Methods:**

This descriptive-analytical cross-sectional study was conducted in Kerman from October to December 2020. The research population included all lactating women who visited health centers affiliated to Kerman University of Medical Sciences to receive health services. The data in this study were collected using a checklist.

**Results:**

A total of 228 mothers who met the inclusion criteria participated in the study. A total of 221 mothers (97.0%) received nonprescribed medications (377 drugs in total). Among 377 nonprescribed medications, 279 drugs (74.0%) could be used while breastfeeding. The three most commonly used nonprescribed medications were acetaminophen tablets (84 [22.3%]), gelofen compound (51 [13.5%]), and adult cold medications. The majority of the mothers, 153 (40.6%), chose nonprescribed medications based on pharmacy staff recommendations. The COVID-19 outbreak was the most common reason for using 246 (65.3%) nonprescribed drugs. There was a statistically significant relationship between the mothers’ education and the accuracy of their information about the side effects of nonprescribed medications use.

**Conclusion:**

The prevalence of self-medication by lactating women during the COVID-19 pandemic was very high. A significant percentage of the mothers did not have correct information about the adverse effect of arbitrary use of nonprescribed drugs on their babies.

## Introduction

According to the World Health Organization (WHO), self-medication refers to the selection and use of medicines to treat self-recognized illnesses or symptoms without consulting a health care provider [1,2]. Self-medication includes the use of over-the-counter (OTC) medications, the overuse of prescribed drugs, the use of drugs remaining from previous prescriptions, and the use of herbal and traditional medicines [3].

The prevalence of self-medication varies in different societies and can be influenced by various factors such as lack of access to healthcare services, excessive distribution of drugs, patients’ attitude toward health care providers, socioeconomic factors, long waiting times at the health facilities, medication expense, education level, age, monthly income, and people’s satisfaction with and beliefs about medicines and diseases [4,5]. Moreover, women are more interested in the arbitrary use of drugs to treat problems such as painful menstruation, menopausal symptoms, mood disorders, and prevention of osteoporosis, as well as problems that occur during pregnancy and lactation [6]. Analgesics, laxatives, vitamins, antibiotics, and sedatives are the most commonly used postpartum drugs [7].

Medication use is common during breastfeeding. For instance, a study on pregnant and lactating women in the United States found that most women used at least one drug and an average of 3.3 drugs during breastfeeding [8]. Another study showed that the prevalence of arbitrary drug use during lactation in the Netherlands was approximately 65%. Besides, 61.2% of the mothers had poor awareness about the correct use of drugs [6]. However, most mothers are concerned about taking medication while breastfeeding and may use information about whether or not to take medications from different sources, including physicians, pharmacists, midwives, spouses, friends and neighbors, or media; they stop breastfeeding in some cases without the desire to do so as a result of incorrect advice from others [9]. Thus, breastfeeding women need correct advice on the use of medications during breastfeeding [10]. It should be noted that the use of drugs during breastfeeding by mothers without a physician’s advice may affect breast milk, or the drug may reach the baby through milk [11].

On the other hand, since the last two years, as the world has been facing the COVID-19 pandemic, there has been a general misconception that the only source available to people to help them is self-care and self-medication [12]. Dissemination of fake news [13] and the existence of concerns and restrictions such as lockdowns and prohibitions on attending very crowded places such as doctors’ offices have promoted the use of self-medication in various groups of society. Furthermore, pregnant and lactating women are much more concerned about the transmission of the COVID-19 disease and even death due to it. These concerns may increase the possibility of arbitrary drug use among women [14].

Considering these factors, the present study aimed to investigate the prevalence of arbitrary drug use and factors affecting it in lactating mothers visiting health centers affiliated to Kerman University of Medical Sciences during the COVID-19 pandemic and to determine the accuracy of the mothers’ information about arbitrary drug use.

## Material and methods

This descriptive-analytical cross-sectional study was conducted in Kerman from October to December 2020. This study was approved by the ethics committee of Kerman University of Medical Sciences under the number IR.KMU. REC.1399.363. Each participant read and signed an informed consent form before beginning the study. The research population included all the breastfeeding women who visited health centers (10 centers in total) affiliated to Kerman University of Medical Sciences to receive health services. Sampling was performed in all the centers. Mothers who were not residents of Kerman, mothers who exclusively used a formula to feed their infants, and those who were barred from breastfeeding their infants due to certain diseases were excluded from the study.

A self-designed checklist was used to collect data. The checklist was designed based on expert opinions of six specialists in this regard. It contained two separate sections. The items in the first section assessed the participants’ demographic characteristics (age of mothers and infants, route of birth delivery, mothers’ education and jobs, and fathers’ jobs and income per month). The first part of the second section evaluated the infant’s diet, such as the use of formula or complementary food in addition to breast milk, and the daily breastfeeding patterns. Then, the participants answered a question in the checklist: ‘Have you used any chemical or herbal medicines during breastfeeding without consulting your doctor?’ A positive answer to this question confirmed the arbitrary use of drug(s) during lactation. The rest of the questions in the second part were completed only by the mothers who were taking medication arbitrarily during breastfeeding. These questions were about the sources of the drug information that convinced the participants to turn to the arbitrary use of drugs, the number of medications used by the mothers, duration of the nonprescribed medication use, time of the drug use during the 18 months after delivery (the first, second, and third six months), common indications for self-medication, the reasons for arbitrary use of the drugs, reading the drugs’ information leaflets before using them, and their effects on the quality and quantity of milk. The mothers were also asked about the secretion of the drug into breast milk, whether the drug use during lactation was permissible or not, routine breastfeeding while taking nonprescribed medications, and the adverse effects of the drug use on the infants during breastfeeding.

The accuracy of the data provided by the mothers about the secretion of the drug into breast milk, routine breastfeeding while taking the nonprescribed medications, and the adverse effects of the drug use on the infants during breastfeeding were checked using the information available in the UptoDate Database (http://www.uptodate.com, accessed 2021), *Neonatal Risk: Drugs in Pregnancy and Lactation: A Reference Guide to Fetal and Neonatal Risk* (Briggs, 11^th^ Edition) [15], and drug information leaflets. Drug information leaflets were used for some drugs that were approved only by the Iran Food and Drug Administration. The information on some drugs was only in UptoDate Database or Briggs. The Anatomical Therapeutic Chemical (ATC) classification system [16] was also used in this study to classify the drugs used by the mothers.

The sample size was estimated to be 96 persons using the following formula: N = z^2^ × p (1-p) / d^2^. In this formula, z (with a 95% confidence interval), p and d were considered as 1.96, 0.5 and 0.1, respectively. According to the previous studies, the prevalence of nonprescribed medications used during lactation was 17% to 75% [8,17,18].

The Statistical Package for Social Science (SPSS) version 23 was used for all the analyses. Descriptive analysis was applied for all the variables. Chi-square and one-way analysis of variance were employed to compare qualitative and quantitative data, respectively. A p-value of less than 0.05 was considered statistically significant.

## Results

A total of 228 mothers who met the inclusion criteria completed the checklist. Among them, 221 mothers used nonprescribed medications. The participants’ mean age was 30.5 ± 5.7 years. Moreover, the mean age of the participants’ children was 10.1 ± 5.2 months. The demographic characteristics of the participating mothers, infants’ diets, and daily breastfeeding patterns are presented in Table 1. In addition, there was not any significant relation between the number of the breastfeeding sessions and the use of nonprescribed medications by mothers (P-value = 0.925). Among 221 mothers using nonprescribed medications, 115 (52.0%), 79 (35.8%), and 27 (12.2%) mothers breastfed their babies 5 to 10, more than 10, and less than 5 times per day, respectively. The data indicated that 221 participants (97.0%) received a total of 377 nonprescribed medications. Among all the 221 mothers, 120 (54.3%), 66 (30.0%), 24 (10.8%), 9 (4.1%), 1 (0.4%), and 1(0.4%) mother(s) used 1, 2, 3, 4, 5, and 6 nonprescribed medication(s), respectively. The three most commonly used nonprescribed medications were acetaminophen tablets (84 [22.3%]), gelofen compound (acetaminophen + ibuprofen + caffeine) (51 [13.5%]), and adult cold medication (acetaminophen with one or more of the following ingredients: diphenhydramine, chlorpheniramine, pseudoephedrine, phenylephrine, guaifenesin, and dextromethorphan) (42 [11.1%]). Other common medications were azithromycin (18 [4.8%]), amoxicillin (16 [4.2%]), ferfolic (14 [3.7%]), calcium (11 [2.9%]) and cefixime (10 [2.7%]). Table 2 shows the nonprescribed medications classification based on the Anatomical Therapeutic Chemical classification system. As shown in Table 2, most nonprescribed medications (45.9%) were used for symptoms related to the nervous system, and 14.1% of the drugs were anti-infectives for systemic use.

**Table 1:** Demographic characteristics of the participating mothers (n = 228), infants’ diets and daily breastfeeding patterns

Characteristics	Frequency (%)
Routes of birth delivery	
Caesarean section	119 (52.2%)
Vaginal delivery	109 (47.8%)
Mothers’ education	
Illiterate	6 (2.6%)
Less than high school diploma	21 (9.2%)
High school diploma	89 (39.0%)
More than high school diploma	112 (49.2%)
Mothers’ jobs	
Housewife	182 (79. 8%)
Employee	41 (18.0%)
University student	1 (0.4%)
Medical staff	4 (1.8%)
Fathers’ jobs	
Unemployed	4 (1.8%)
Self-employed	155 (68.0%)
Employee	63 (27.6%)
University student	1 (0.4%)
Medical staff	5 (2.2%)
Income per month (dollar)	
Less than 70	41 (18.0%)
70-170	134 (58.8%)
More than 170	53 (32.2%)
Infants’ diets	
Exclusive breastfeeding	49 (21.5%)
Breastfeeding plus supplementary food	155 (68.0%)
Breastfeeding plus infant formula	8 (3.5%)
Breastfeeding plus infant formula plus	16 (7.0%)
supplementary food	
Breastfeeding (times per day)	
Less than 5 times	28 (12.3%)
5-10 times	119 (52.2%)
More than 10 times	81 (35.5%)

**Table 2:** Nonprescribed medications classification based on the Anatomical Therapeutic Chemical classification system (N = 377)

Anatomical Therapeutic Chemical classification system	Frequency (%)
Alimentary tract and metabolism	45 (11.9%)
Blood and blood-forming organs	14 (3.7%)
Cardiovascular system	2 (0.5%)
Genito-urinary system and sex hormones	34 (9.0%)
Anti-infectives for systemic use	53 (14.1%)
Musculoskeletal system	32 (8.5%)
Nervous system	173 (45.9%)
Anti-parasitic products, insecticides, and repellents	4 (1.1%)
Respiratory system	19 (5.0%)
Sensory organs	1 (0.30%)
Total	377 (100%)

The majority of the mothers, 153 (40.6%), chose nonprescribed medications based on pharmacy staff recommendations. Some mothers chose more than one source of drug information for self-medication (for 14 [3.7%] drugs). Most of the mothers took nonprescribed medications during the first six months after delivery. Some mothers chose more than one period of time for self-medication (for 85 [22.5%] drugs). The most common problems leading to the use of nonprescribed medications by the participants were migraines and pain (37.4%) and respiratory problems (34.2%). The majority of the mothers, 293 (77.7%), used nonprescribed drugs for less than one week. Table 3 presents the source of the drug information for self-medication, time of the nonprescribed medication use, indications for self-medication, and duration of the nonprescribed medication use.

**Table 3:** Nonprescribed medications use data (N = 377)

Drug use data	Categories	Frequency (%)
Source of drug	Family members and friends	73 (19.3%)
information for self-medication	Internet and online social media	12 (3.2%)
	Pharmacy staff	153 (40.6%)
	Previous experiences	140 (37.1%)
	Health centers	13 (3.4%)
Duration of the	Less than one week	293 (77.7%)
nonprescribed medication use	One week to one month	54 (14.3%)
	More than one month	30 (8.0%)
Time of the drug	The first six months after	225 (60.7%)
use during the	delivery	
18 months after delivery	The second six months after delivery	194 (51.4%)
	The third six months after	52 (13.7%)
	delivery	
Common indica-	Digestive problems	9 (2.4%)
tions medication for self-	Urinary problems	7 (1.9%)
	Migraine and pain	141 (37.4%)
	Problems with milk volume	23 (6.1%)
	Respiratory problems	129 (34.2%)
	Anaemia	15 (4.0%)
	Tooth infection	14 (3.7%)
	Abscesses and breast infec-	3 (0.8%)
	tions	
	Uterine infection	6 (1.6%)
	Other	30 (8.0%)

Also, the questions and mothers’ answers regarding the nonprescribed medications use during breastfeeding (the second part of the checklist) are shown in Table 4. As demonstrated in this table, the participants read 118 (31.3%) drug leaflets while using the nonprescribed drugs. They did not know about the effect of 292 (77.5%) nonprescribed drugs on breast milk. On the other hand, the participants continued breastfeeding routinely while taking 363 (96.3%) nonprescribed drugs. It was also shown that the participants were aware of 286 (75.9%) nonprescribed drugs that enter breast milk. They also thought that 209 (55.4%) nonprescribed drugs were allowed during breastfeeding.

**Table 4:** Questions and mothers’ answers about nonprescribed medications (N = 377) use during breastfeeding

Questions	answers	Frequency (%)
Did you read the drug information leaflet before using the non-prescribed	Yes	118 (31.3%)
medication?	No	259 (7/68%)
Did the drug have any effects on the	Yes	74 (19.6%)
quality and quantity of your milk?	No	292 (77.5%)
	Did not notice	11 (2.9%)
Did you breastfeed as usual while	Yes	363 (96.3%)
taking the nonprescribed medication?	No	14 (3.7%)
	Yes	286 (75.9%)
	No	30 (8.0%)
Is the used drug secreted into breast milk?	Do not know	61 (16.2%)
Is the drug use during lactation	Yes	209 (55.4%)
permissible?	No	28 (7.4%)
	Do not know	140 (37.1%)
Does the used drug have adverse	Yes	20 (5.3%)
effects on infants?	No	343 (91.0%)
	Do not know	14 (3.7%)

The COVID-19 outbreak was the most common reason for using 246 (65.3%) nonprescribed drugs. Reasons for using the nonprescribed medications are presented in Table 5. Some mothers chose more than one reason.

**Table 5:** Reasons for using nonprescribed medications (N = 377)

Reasons	Frequency (%)
COVID-19 outbreak	246 (65.3%)
Financial problems and cost saving	75 (19.9%)
Unseriousness of the disease from the mothers’ perspectives	114 (30.2%)
Consulting with close relatives who are physicians	14 (3.7%)
The mother or father is a physician or a therapist	16 (4.2%)
Absence of health insurance	4 (1.1%)
Consulting with health center staff and care providers	5 (1.3%)
Anti-parasitic products, insecticides, and repellents	4 (1.1%)
Poor health service provision due to living in a deprived area	1 (0.3%)
Easier access to a pharmacy than to a doctor	52 (13.8%)

Table 6 demonstrates the accuracy of the mothers’ information about the nonprescribed medications during breastfeeding, including the side effects, routine breastfeeding, and secretion into breast milk. These mothers had correct information about the side effects of 212 (56.2%) nonprescribed medications, routine breastfeeding while taking 278 (73.7%) nonprescribed medications, and secretion of 261 (69.2%) of these medications into breast milk.

**Table 6:** Accuracy of the mothers’ information answers aboutnonprescribed medications (N = 377) use during breastfeeding

The mothers’ information about nonprescribed medications during breastfeeding		Frequency (%)
		
The accuracy of the mothers’	Accurate	212 (56.2%)
information about the side effects	information	
of nonprescribed medications	Inaccurate	131 (34.7%)
	information	
	The mothers	14 (3.7%)
	did not have	
	any information	
	Not enough	20 (5.3%)
	data available	
The accuracy of the mothers’	Accurate	278 (73.7%)
information about routine breast-	information	
feeding while taking nonprescribed medications	Inaccurate information	73 (19.4%)
	Not enough	26 (6.9%)
	data available	
The accuracy of the mothers’	Accurate	261 (69.2%)
information about secretion of	information	
nonprescribed medications into breast milk	Inaccurate information	31 (8.2%)
	The mothers	41 (10.9%)
	did not have any	
	information	
	Not enough	44 (11.7%)
	data available	

Among 377 nonprescribed medications, 279 (74.0%) could be used while breastfeeding. Table 7 shows the classification of nonprescribed medications (n = 377) during breastfeeding based on UptoDate Database, drug information leaflets, and Briggs.

**Table 7:** Classification of nonprescribed medications (n = 377) during breastfeeding based on UptoDate Database, drug information leaflets, and Briggs

Classification	Frequency (%)
Compatible	279 (74.0%)
No human data – probably compatible	61 (16.2%)
Breastfeeding is contraindicated by the manufacturer	18 (4.8%)
Limited human data – probably compatible	6 (1.6%)
Breastfeeding is allowed only upon the physician’s advice	1 (0.3%)
Breastfeeding is not recommended by the manufacturer	10 (2.7%)
Limited human data – potential toxicity	2 (0.5%)

Also, there was a statistically significant relationship between the participants’ education and their correct information about the side effects of nonprescribed medications (P-value = 0.0001). The more educated mothers were, the more accurate their information was. However, the mothers’ jobs (P-value = 0.214) and age (P-value = 0.760), and fathers’ jobs (P-value = 0.742) and household income (P-value = 0.098) had no significant correlations with the mothers’ correct information about the side effects of nonprescribed medications.

Furthermore, the mothers’ jobs (P-value = 0.019) were significantly associated with the correct approach to routine breastfeeding. However, the mothers’ education (P-value = 0.533) and age (P-value = 0.355), and fathers’ jobs (P-value = 0. 905) and household income (P-value = 0.377) were not significantly associated with this approach.

Moreover, the mothers’ education (P-value = 0.0001) and age (P-value = 0.007), and fathers’ jobs (P-value = 0.008) and household income (P-value = 0.001) had significant effects on the accuracy of the mothers’ information about the secretion of the drug into breast milk, while the mothers’ job (P-value = 0.372) had no significant effect on it.

## Discussion

This study examined the use of nonprescribed medications by breastfeeding mothers visiting health centers affiliated to Kerman University of Medical Sciences during the COVID-19 pandemic. The results showed that 221 of the participating mothers took 377 nonprescribed medications. The most commonly used medications were acetaminophen, gelofen compound, and cold medicines. The highest number of drugs (161) was taken in the first six months after delivery. The most common problems and illnesses that led mothers to use nonprescribed medications during breastfeeding were migraines, pain, and respiratory problems. It was also shown that these mothers had accurate information about the side effects of the 212 nonprescribed medications on their infants. Furthermore, of the 377 medications, 279 could be used during breastfeeding. Correspondingly, most of the nonprescribed medications (74.0%) could be used safely when a mother breastfed, and discontinuing breastfeeding because of the medications was not necessary.

The results showed there was a significant relationship between the mothers’ education and their correct information about the side effects of nonprescribed medications. The mothers’ education, fathers’ jobs and income, and mothers’ age had significant effects on the accuracy of the mothers’ information about the secretion of the drug into breast milk. The mothers’ jobs were significantly associated with the correct approach to routine breastfeeding.

Previous studies have investigated self-medication by lactating women. For instance, Lutz et al. showed that 89.2% of the mothers in their study used drugs during lactation, of whom only 2.8% self-medicated. Acetaminophen was the most widely used drug, followed by iron sulfate and desogestrel. The maximum duration of medication was up to seven days. A comparison of the demographic factors indicated that only the mothers’ education was directly related to drug use during breastfeeding [19]. In another study, Suksamarnwong et al. reported that 31.4% of the mothers in their study used drugs during the first month postpartum, and no advice was given to 63.2% of them about drug use during breastfeeding. The primary source of drugs was pharmacies (79.7%) and 85.5% of the mothers continued breastfeeding while taking drugs. Also, the authors reported a positive relationship between the mothers’ education and drug use during breastfeeding [20].

Also, Al-Sawalha et al. found that approximately 17% of the breastfeeding mothers used OTC medications and 7% used both OTC and prescribed medications. Most of the drugs that they took were painkillers and antibiotics. Half of the mothers were aware of the dangers of taking drugs for their babies. Two-thirds of the mothers read the drug information leaflets before taking their medications. Other sources of information about the medicines used by the mothers were health care providers, relatives, and the Internet. Furthermore, 49.1% of the mothers reported that they took the drugs during breastfeeding, but only 5.2% of them noticed side effects such as diarrhea or decreased activity in their infants [18]. The last finding was closely in line with the results of the present study. In a study, Mota et al. found that 19% of the mothers self-medicated during breastfeeding. The medications used were nonsteroidal anti-inflammatory drugs, contraceptives, antibiotics, and antihypertensive drugs that were taken mostly for headaches and postoperative infections [21]. Chaves et al. showed that 52.4% of the mothers used self-medication during breastfeeding. The most commonly used drugs were analgesics, antipyretics, and nonsteroidal anti-inflammatory drugs. The results also showed that there was no relationship between any of the demographic variables and self-medication [8].

In another study, de Waard et al. showed that 75.9% of the mothers used OTC drugs, mostly painkillers and vitamins, during breastfeeding. Fifteen (11.3%) of the mothers used paracetamol plus codeine or morphine, which posed a (potential) risk to the baby; the rest of the drugs were safe or potentially safe [17]. Also, Jones and Brown showed that 53.6% of the women who breastfed their infants bought the drugs themselves. Most of the drugs used by them were painkillers and antibiotics [22]. Stultz et al. found that the drugs most commonly used by the breastfeeding women were multivitamins, nonsteroidal anti-inflammatory drugs, and acetaminophen [23].

There are some similarities between the results reached in the above-mentioned studies and the ones obtained in the current study regarding the most common medications used and the indications for self-medication. But the most common differences in the results are related to the prevalence of self-medication.

The contradictory results reported in the previous studies and the present one could be attributed to the participants’ characteristics and the time of data collection. It seems that cultural and socioeconomic issues in different societies can influence decisions of people about self-medication [24]. It was reported that there was a relatively higher prevalence of self-medication among the Iranian people compared to that in other countries [25]. Also, financial issues are one of the main factors leading to self-medication in Iran (lower-middle income economy). As reported previously, the most important reason for self-medication in Iran was lack of health insurance [26]. Furthermore, the COVID-19 outbreak and the associated problems caused by this disease, especially people’s concerns about being in crowded places such as doctors’ offices or specialised clinics, made people self-medicate more frequently than before [27, 28]. Increasing awareness of breastfeeding mothers about the risks of arbitrary use of drugs through educational programs is recommended [6, 17]. The major limitation of this study was its restriction to one city in Iran. For more reliable assessments, it would be better to perform such studies in several cities of Iran.

## Conclusion

The prevalence of self-medication by lactating women during the COVID-19 pandemic was very high. And a significant percentage of mothers did not have correct information about the adverse effect of arbitrary use of drugs on their babies.

## Keypoints

The prevalence of self-medication by lactating women during the COVID-19 pandemic was very high.

A significant percentage of mothers did not have correct information about the adverse effect of arbitrary use of drugs on their babies.

There was a significant relationship between the mothers’ education and their correct information about the side effects of nonprescribed medications on their infants.
